# Supplementary material for: Insulin-Degrading Enzyme Interacts with Mitochondrial Ribosomes and Respiratory Chain Proteins
Source: Biomolecules. 2023 May 26;13(6):890. doi: 10.3390/biom13060890 (PMC10296325; doi:10.3390/biom13060890)
Supplement: Supplementary file 1 [file biomolecules-13-00890-s001.zip › biomolecules-2390776-supplementary.pdf]

Table S1: Primers and reagents used

Primers

Mito-IDE forward (**XhoI**)	5′ GAAGT**CTCGAG**GCCACCATGCGGTACCGGCTAGCGG 3′
Mito-IDE reverse (**EcoRI**)	′5′ CCACGCTT**GAATTC**GGTCATG 3′ ′
Linker Forward (**SmaI**)	5′AGAG**CCCGGG**AGCGCCGGCAGCGCCGGCAAG 3′
TurboID Reverse (**MluI**)	′5′ GCCAGA**ACGCGT**CCCGTCCAG 3′

**Reagent or Resource**	**Source**	**Identifier**
**Antibody**		
Anti-IDE (polyclonal)	Abcam	ab32216
Anti-IDE (monoclonal)	Santa Cruz Biotechnology	sc-393887
V5 Tag (monoclonal)	Invitrogen	R96025
TOM20 (polyclonal)	Proteintech	11802-1-AP
Anti-OVA (polyclonal)	Polysciences	23744-5
Anti-rabbit IgG, HRP linked	Cell signaling Technology	7074
Anti-mouse IgG, HRP linked	Cell signaling Technology	7076
**Chemicals**		
NucleoBond Xtra Midi EF	Macherey-Nagel	740420.10
DMEM		
Biotin	Sigma-Aldrich	B4501
Complete EDTA-free protease inhibitors	Roche Diagnostic	11873580001
NuPage 4-12% Bis-Tris Gel	Thermo Fisher Scientific	NP0322BOX
iBlot2 PVDF Mini Stacks	Thermo Fisher Scientific	IB24002
Streptavidin HRP	BD Biosciences	554066
SuperSignalTM West Pico PLUS Chemiluminescent	Thermo Fisher Scientific	34580
BLUeye Prestained Protein Ladder	Sigma	94964
NuPage reducing agent	Thermo Fisher Scientific	NP0004
4x Laemmli sample buffer	BioRad	1610747
VECTASHIELD® PLUS Antifade Mounting Medium	Eurobio Scientific	H-1900-10
**Software**		
Icy	Icy community platform	https://icy.bioimageanalysis.org
String	String consortium	https://string-db.org/
Metascape	Metascape	https://metascape.org
Affinity designer 2	Serif Ltd	https://affinity.serif.com

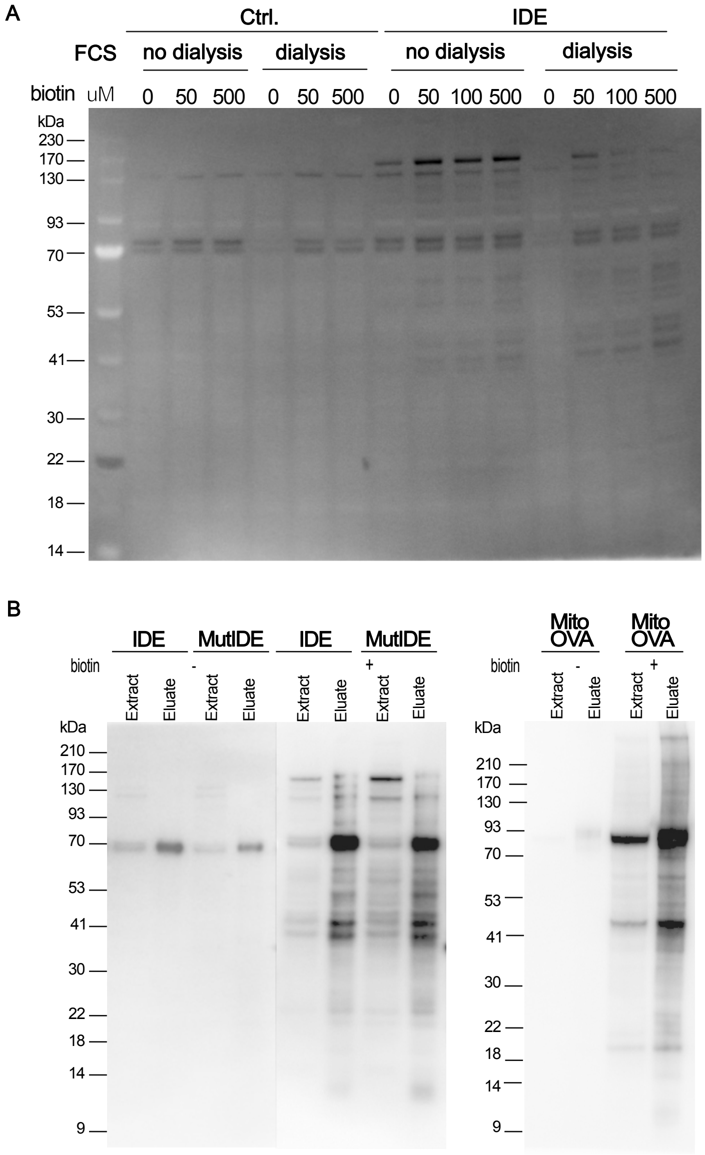

Figure S1. Characterization of biotinylation activity TurboID fusion proteins.
(**A**) Hek293T cells stably expressing IDE-TurboID fusion proteins and untransduced cells (Ctrl) were cultured in presence of non-dialyzed or dialyzed FCSbefore treatment with various concentrations of biotin for 10 min. Whole-cell lysates (10 μg) were analyzed by streptavidin-HRP immunoblotting. (**B**) Immunoblot analysis of biotinylated proteins in total extracts and after enrichment using streptavidin beads, of cells expressing IDE (left) or OVA (right) fusion proteins and incubated for 10 min with 50 μM biotin or buffer control. One representative out of four independent experiments is shown.

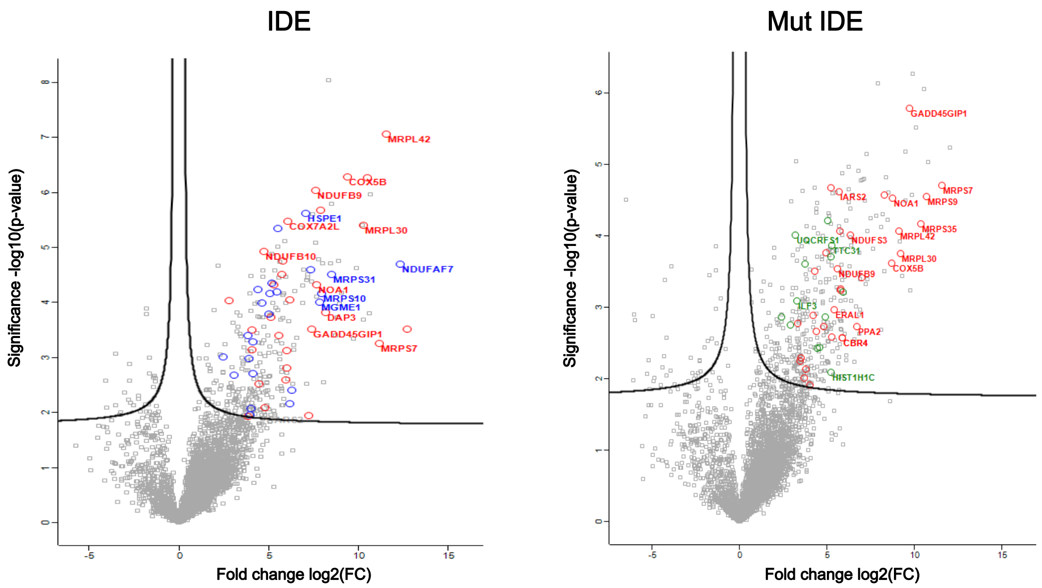

Figure S2. Volcano plots of IDE and MutIDE proteomic data sets.
Scatterplots of biotinylated proteins purified from HEK cells expressing IDE- and MutIDE fusion proteins treated with 50 μM biotin for 10min; data are based on four independent replicates. Mass spectrometry identified 3779 proteins (FDR=0.05). Among these, 62 proteins (IDE) and 53 proteins (MutIDE), respectively, were enriched significantly relative to both the spatial compartmentalization control (OVA fusion protein) and to control cells not incubated with biotin (n=4 replicates for each condition). Significant enriched proteins common to IDE and MutIde are marked with red circles, proteins enriched only in IDE cells in blue circles, and proteins enriched only in MutIDE in green circles.

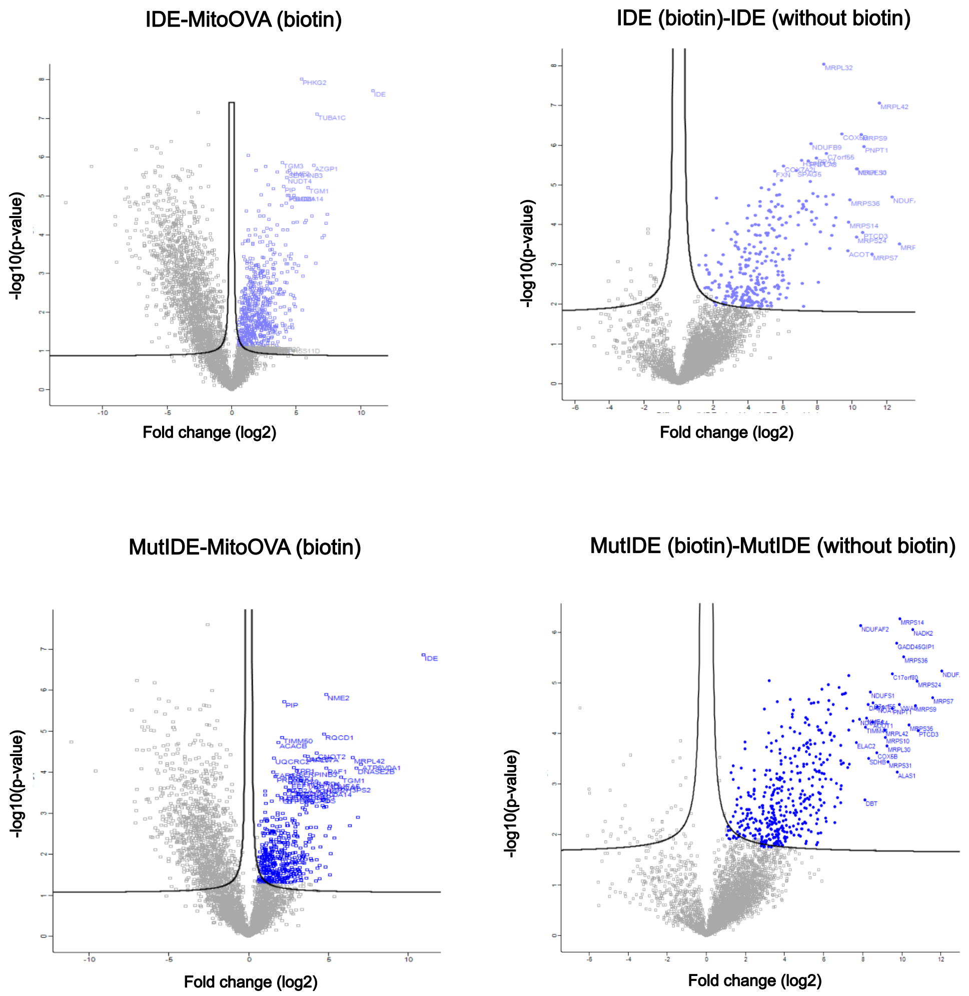

Figure S3. Volcano plots of IDE and MutIDE proteomic data sets with comparison to single controls. Data are represented as in Figure S2 and show scatterplots of proteins from cells expressing IDE wt (top row) or MutIDE fusion proteins with comparison to OVA controls (left column) or IDE controls without biotin (right).

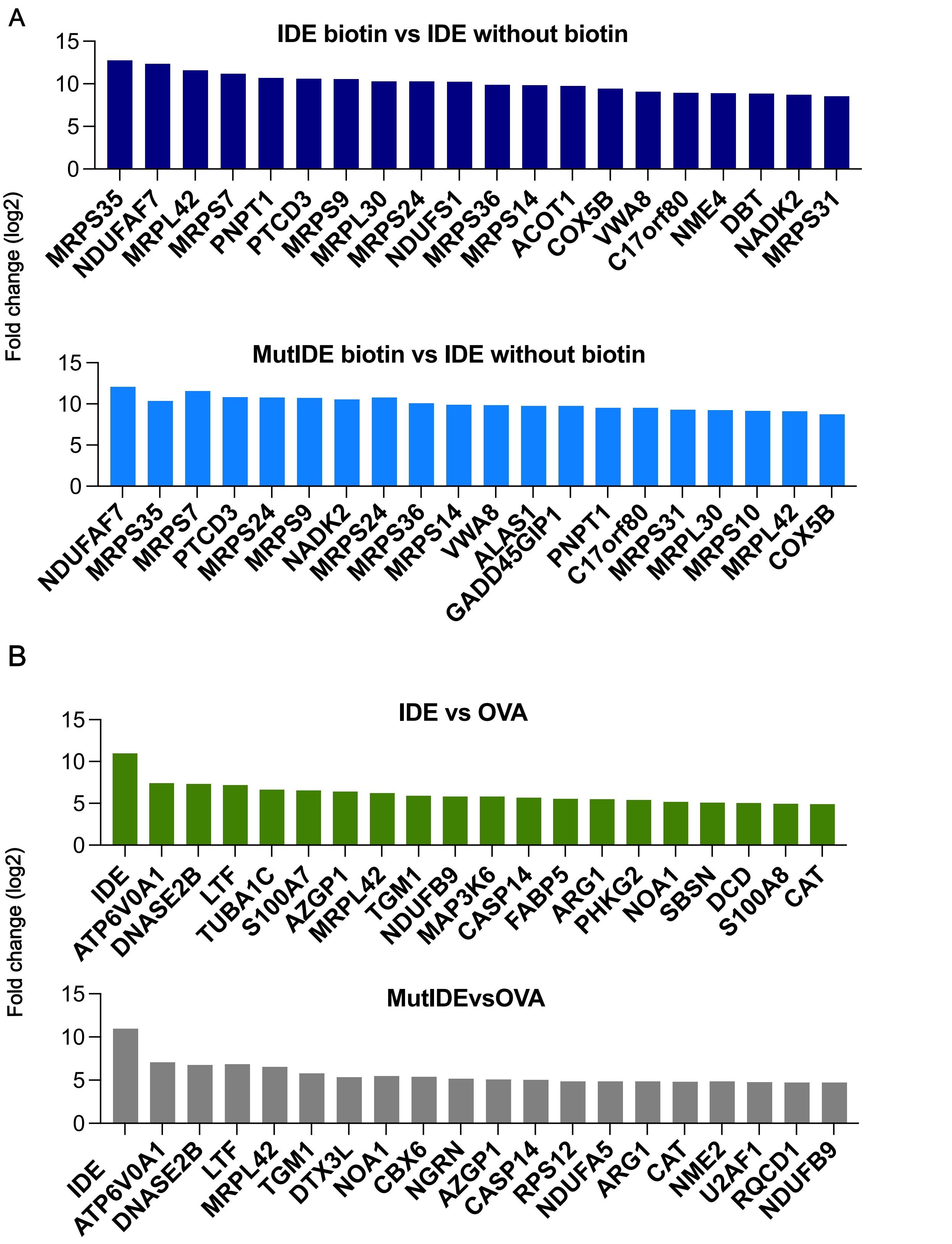

Figure S4. Top 20 enriched proteins interacting with wt or mutant IDE compared either to OVA or to no-biotin controls.

(**A**) Interactomes of wt (top) and protease-dead (bottom) IDE identified by comparing samples with and without biotin addition. Minimum fold change was 8.5 (top) and 8.7, respectively. (**B**) Interactomes of wt (top) and protease-dead (bottom) IDE identified by comparing IDE samples with biotin to OVA samples with biotin. Minimum fold change was 4.9 (top) and 4.7, respectively.

A

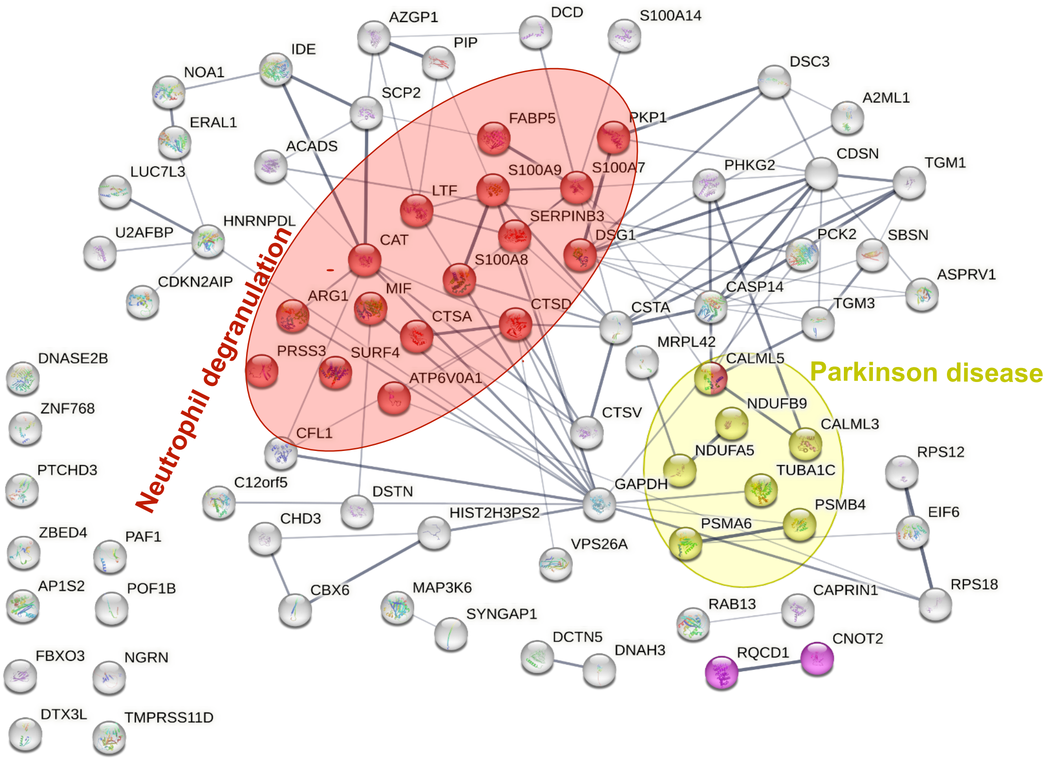

B

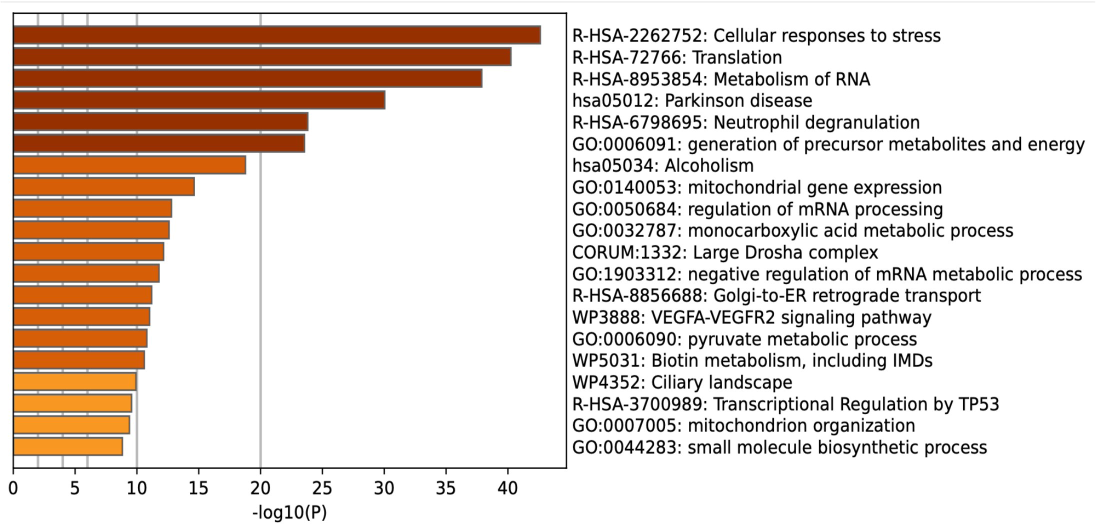

Figure S5. Functional enrichment analysis of the wt IDEinteractome obtained by comparing cells expressing IDE and OVA incubatedwith biotin. (A) Functionally associated protein networks in the IDE interactome as identified by STRING analysis. Proteins belonging to the same biological system are labeled with identical color. (C) Top non-redundant enrichment clusters in the IDE interactome, as identified by Metascape. The color scale represents statistical significance levels expressed as -log10.
